# *cis*-Aqua­bis(2,4-dichloro-6-formyl­phenolato-κ^2^ *O*,*O*′)(*N*,*N*-dimethyl­formamide-κ*O*)nickel(II)

**DOI:** 10.1107/S1600536808022939

**Published:** 2008-07-26

**Authors:** Fa-Yun Chen, Shu-Hua Zhang, Chen-Min Ge

**Affiliations:** a Department of Chemistry, Shangrao Normal University, Shangrao, Jiangxi 334001, People’s Republic of China; b Key Laboratory of Nonferrous Metal Materials and Processing Technology, Department of Materials and Chemical Engineering, Guilin University of Technology, Ministry of Education, Guilin 541004, People’s Republic of China

## Abstract

In the title compound, [Ni(C_7_H_3_Cl_2_O_2_)_2_(C_3_H_7_NO)(H_2_O)], the Ni^II^ ion is coordinated by four O atoms from two bidentate 2,4-dichloro-6-formyl­phenolate ligands, one O atom from a water ligand and one O atom from a dimethyl­formamide ligand in a slightly distorted octa­hedral environment. In the crystal structure, centrosymmetric dimers are formed though O—H⋯O and O—H⋯Cl hydrogen bonds; π–π stacking inter­actions, with a centroid–centroid distance of 3.796 (2) Å, are also found.

## Related literature

For related literature, see: Cohen *et al.* (1964 ▶); Desiraju (1989 ▶); Mathews & Manohar (1991 ▶); Zaman *et al.* (2004 ▶); Zhang *et al.* (2007 ▶); Zordan *et al.* (2005 ▶).
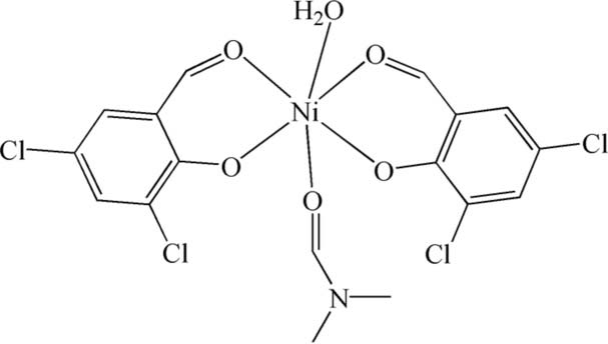


## Experimental

### 

#### Crystal data

[Ni(C_7_H_3_Cl_2_O_2_)_2_(C_3_H_7_NO)(H_2_O)]
*M* *_r_* = 529.81
Monoclinic, 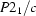

*a* = 10.404 (2) Å
*b* = 9.6130 (19) Å
*c* = 22.161 (4) Å
β = 92.44 (3)°
*V* = 2214.4 (8) Å^3^
*Z* = 4
Mo *K*α radiation
μ = 1.39 mm^−1^
*T* = 293 (2) K
0.48 × 0.40 × 0.35 mm

#### Data collection

Bruker SMART CCD diffractometer
Absorption correction: multi-scan (*SADABS*; Sheldrick, 1996 ▶) *T*_min_ = 0.555, *T*_max_ = 0.642
10765 measured reflections
3969 independent reflections
3010 reflections with *I* > 2σ(*I*)
*R*_int_ = 0.036

#### Refinement

*R*[*F* ^2^ > 2σ(*F* ^2^)] = 0.042
*wR*(*F* ^2^) = 0.111
*S* = 1.07
3969 reflections
266 parameters
H atoms treated by a mixture of independent and constrained refinement
Δρ_max_ = 0.59 e Å^−3^
Δρ_min_ = −0.37 e Å^−3^

### 

Data collection: *SMART* (Bruker, 2004 ▶); cell refinement: *SAINT* (Bruker, 2004 ▶); data reduction: *SAINT*; program(s) used to solve structure: *SHELXS97* (Sheldrick, 2008 ▶); program(s) used to refine structure: *SHELXL97* (Sheldrick, 2008 ▶); molecular graphics: *SHELXTL* (Sheldrick, 2008 ▶); software used to prepare material for publication: *SHELXTL*.

## Supplementary Material

Crystal structure: contains datablocks global, I. DOI: 10.1107/S1600536808022939/lh2644sup1.cif

Structure factors: contains datablocks I. DOI: 10.1107/S1600536808022939/lh2644Isup2.hkl

Additional supplementary materials:  crystallographic information; 3D view; checkCIF report

## Figures and Tables

**Table 1:** Selected bond lengths (Å)

Ni1—O3	2.041 (2)
Ni1—O1	2.041 (2)
Ni1—O2	2.061 (3)
Ni1—O4	2.070 (3)
Ni1—O5	2.148 (3)
Ni1—O6	2.150 (3)

**Table 2:** Hydrogen-bond geometry (Å, °)

*D*—H⋯*A*	*D*—H	H⋯*A*	*D*⋯*A*	*D*—H⋯*A*
O6—H6*A*⋯O1^i^	0.82	1.91	2.714 (3)	168
O6—H6*B*⋯O3^i^	0.83 (4)	2.17 (4)	2.850 (4)	139 (4)
O6—H6*B*⋯Cl3^i^	0.84 (4)	2.67 (4)	3.374 (3)	143 (4)

Symmetry code: (i) 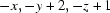.

**Fig. 1.**
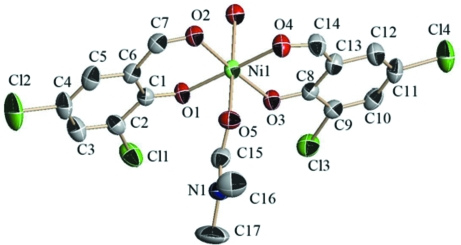
A view of (I), showing 30% probability displacement ellipsoids. Hydrogen atoms are omitted.

**Fig. 2.**
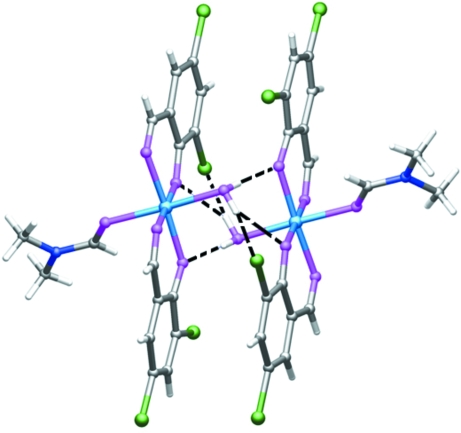
The dimer of (I), Dashed lines indicate hydrogen bonds.

**Fig. 3.**
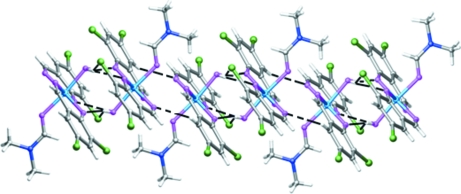
1-D chain of (I), Dashed lines indicate hydrogen bonds.

## supplementary crystallographic information

### Comment

Halogens have a ubiquitous presence in both inorganic and organic chemistry. Schiff bases of chloro substituents on aromatic groups have aroused increasing interest in recent years because these halogenated compounds are an attractive target for use in supramolecular chemistry and crystal engineering wherein the halogen atoms are directly involved in forming intermolecular interactions (Cohen *et al.*, 1964; Zordan *et al.*, 2005; Desiraju, 1989; Zaman *et al.*, 2004; Zhang *et al.*, 2007). The title compound, (I), contains the dichloride ligand 3,5-Dichloro-2-hydroxy-benzaldehyde, with two Cl atoms accessible at the periphery of each ligand.

In the molecular structure of (I), the Ni^II^ ion is coordinated by four O atoms from two bidentate 3,5-Dichloro-2-hydroxy-benzaldehyde ligands, one O atom from a H_2_O ligand and one O atom from a *N*,*N*'-dimethylformamide ligand forming a slightly distorted octahedral geometry (Fig. 1). In the crystal structure O—H···O and O—H···Cl hydrogen bonds (see Table 2) result in the formation of a centrosymmetric dimer (Fig. 2). Within the dimer, there are π–π stacking interactions between the C1–C6 and C8–C13(-x, 2-y, 1-z) rings with centroid···centroid distance of 3.796 (2) Å and interplanar distance of 3.59 Å giving an offset angle of 3.5°. In the crystal structure, dimers are further linked through weak intermolecular C—H···O hydrogen bonds (Fig. 3) (C5—H5A···O4^ii^, 3.454 Å, symmetry code: (ii) 1 + *x*, 2 - *y*, 1 + *z*).

### Experimental

A ethanol solution (30 ml) containing 3,5-Dichloro-2-hydroxy-benzaldehyde (0.191 g, 1 mmol) was dropwise added to an aqueous solution containing amino-methanesulfonic acid (0.111 g, 1 mmol) and sodium hydroxide (0.040 g, 1 mmol) with stirred during 10 min. After stirring for 1 h, an aqueous solution of Nickel chloride (0.237 g, 1 mmol) was added to the resulting solution and stirred for 2 h. The green solid compound was separated out and dissolved by *N*,*N*-Dimethylformamide, then the green solution was filtrated. After 10 days, green crystals were produced from the filtrate (yield: 65.3%, based on Ni).

### Refinement

H atoms were positioned geometrically and were treated as riding atoms, with C—H distances of 0.93–0.96 Å and *U*_iso_(H) = 1.2*U*_eq_(C), and with and O—H distance of 0.82 Å and *U*_iso_(H) = 1.5*U*_eq_(O) for H6A. Atom H6B was refined independently with an isotropic displacement parameter.

### Crystal data

**Table:**

[Ni(C_7_H_3_Cl_2_O_2_)_2_(C_3_H_7_N_1_O_1_)(H_2_O_1_)]	*F*_000_ = 1072
*M**_r_* = 529.81	*D*_x_ = 1.589 Mg m^−^^3^
Monoclinic, *P*2_1_/*c*	Mo *K*α radiation λ = 0.71073 Å
Hall symbol: -P 2ybc	Cell parameters from 3969 reflections
*a* = 10.404 (2) Å	θ = 1.8–25.2º
*b* = 9.6130 (19) Å	µ = 1.39 mm^−^^1^
*c* = 22.161 (4) Å	*T* = 293 (2) K
β = 92.44 (3)º	Block, green
*V* = 2214.4 (8) Å^3^	0.48 × 0.40 × 0.35 mm
*Z* = 4	

### Data collection

**Table:**

Bruker SMART CCD diffractometer	3969 independent reflections
Radiation source: fine-focus sealed tube	3010 reflections with *I* > 2σ(*I*)
Monochromator: graphite	*R*_int_ = 0.036
Detector resolution: 0 pixels mm^-1^	θ_max_ = 25.2º
*T* = 293(2) K	θ_min_ = 1.8º
ω scans	*h* = −12→12
Absorption correction: multi-scan(SADABS; Sheldrick, 1996)	*k* = −11→11
*T*_min_ = 0.555, *T*_max_ = 0.642	*l* = −23→26
10765 measured reflections	

### Refinement

**Table:**

Refinement on *F*^2^	Secondary atom site location: difference Fourier map
Least-squares matrix: full	Hydrogen site location: inferred from neighbouring sites
*R*[*F*^2^ > 2σ(*F*^2^)] = 0.042	H atoms treated by a mixture of independent and constrained refinement
*wR*(*F*^2^) = 0.111	*w* = 1/[σ^2^(*F*_o_^2^) + (0.0455*P*)^2^ + 2.189*P*] where *P* = (*F*_o_^2^ + 2*F*_c_^2^)/3
*S* = 1.07	(Δ/σ)_max_ < 0.001
3969 reflections	Δρ_max_ = 0.59 e Å^−^^3^
266 parameters	Δρ_min_ = −0.37 e Å^−^^3^
Primary atom site location: structure-invariant direct methods	Extinction correction: none

### Special details

**Table:**

Geometry. All e.s.d.'s (except the e.s.d. in the dihedral angle between two l.s. planes) are estimated using the full covariance matrix. The cell e.s.d.'s are taken into account individually in the estimation of e.s.d.'s in distances, angles and torsion angles; correlations between e.s.d.'s in cell parameters are only used when they are defined by crystal symmetry. An approximate (isotropic) treatment of cell e.s.d.'s is used for estimating e.s.d.'s involving l.s. planes.
Refinement. Refinement of *F*^2^ against ALL reflections. The weighted *R*-factor *wR* and goodness of fit *S* are based on *F*^2^, conventional *R*-factors *R* are based on *F*, with *F* set to zero for negative *F*^2^. The threshold expression of *F*^2^ > σ(*F*^2^) is used only for calculating *R*-factors(gt) *etc*. and is not relevant to the choice of reflections for refinement. *R*-factors based on *F*^2^ are statistically about twice as large as those based on *F*, and *R*-factors based on ALL data will be even larger.

### Fractional atomic coordinates and isotropic or equivalent isotropic displacement parameters (Å^2^)

**Table:**

	*x*	*y*	*z*	*U*_iso_*/*U*_eq_	
Ni1	0.20449 (4)	0.95403 (5)	0.46317 (2)	0.03298 (15)	
Cl3	−0.19959 (10)	0.74563 (12)	0.37793 (5)	0.0551 (3)	
Cl1	0.04788 (11)	0.62415 (13)	0.61561 (5)	0.0620 (3)	
Cl4	−0.17775 (12)	1.10954 (17)	0.18744 (5)	0.0754 (4)	
Cl2	0.48052 (13)	0.70149 (19)	0.75310 (6)	0.0915 (5)	
O2	0.3577 (2)	1.0446 (3)	0.51032 (11)	0.0395 (6)	
O1	0.1544 (2)	0.8520 (3)	0.53947 (10)	0.0363 (6)	
O4	0.2662 (2)	1.0551 (3)	0.38720 (11)	0.0439 (7)	
O6	0.1041 (2)	1.1430 (3)	0.48293 (13)	0.0384 (6)	
H6A	0.0272	1.1332	0.4743	0.058*	
O3	0.0510 (2)	0.8740 (3)	0.41437 (10)	0.0374 (6)	
O5	0.3235 (2)	0.7792 (3)	0.44247 (12)	0.0437 (7)	
C1	0.2302 (3)	0.8212 (4)	0.58538 (15)	0.0335 (8)	
C7	0.4025 (3)	1.0013 (4)	0.55949 (17)	0.0393 (9)	
H7A	0.4775	1.0444	0.5742	0.047*	
C8	0.0074 (3)	0.9226 (4)	0.36312 (16)	0.0335 (8)	
C9	−0.1159 (3)	0.8770 (4)	0.33844 (16)	0.0381 (9)	
C5	0.4263 (4)	0.8523 (5)	0.64989 (17)	0.0485 (11)	
H5A	0.5037	0.8981	0.6584	0.058*	
C11	−0.1049 (4)	1.0351 (5)	0.25254 (17)	0.0491 (11)	
C4	0.3865 (4)	0.7480 (5)	0.68905 (18)	0.0536 (12)	
C13	0.0725 (4)	1.0237 (4)	0.32556 (16)	0.0405 (9)	
C12	0.0161 (4)	1.0781 (5)	0.27125 (17)	0.0491 (11)	
H12A	0.0604	1.1422	0.2486	0.059*	
C6	0.3515 (3)	0.8893 (4)	0.59776 (16)	0.0363 (9)	
C3	0.2688 (4)	0.6781 (5)	0.67818 (19)	0.0531 (11)	
H3A	0.2423	0.6093	0.7044	0.064*	
C14	0.2000 (4)	1.0767 (4)	0.34105 (18)	0.0456 (10)	
H14A	0.2360	1.1341	0.3125	0.055*	
C10	−0.1696 (4)	0.9316 (4)	0.28593 (17)	0.0450 (10)	
H10A	−0.2501	0.9005	0.2719	0.054*	
C2	0.1946 (4)	0.7137 (4)	0.62829 (18)	0.0424 (9)	
N1	0.3665 (4)	0.5364 (4)	0.43658 (16)	0.0520 (9)	
C15	0.2951 (4)	0.6490 (5)	0.45053 (17)	0.0454 (10)	
H15A	0.2169	0.6310	0.4678	0.054*	
C16	0.4864 (5)	0.5594 (6)	0.4064 (3)	0.0785 (16)	
H16A	0.5039	0.6574	0.4049	0.118*	
H16B	0.4787	0.5230	0.3661	0.118*	
H16C	0.5554	0.5131	0.4284	0.118*	
C17	0.3215 (7)	0.3854 (5)	0.4433 (3)	0.102 (2)	
H17A	0.2417	0.3840	0.4635	0.152*	
H17B	0.3852	0.3339	0.4666	0.152*	
H17C	0.3093	0.3440	0.4041	0.152*	
H6B	0.098 (4)	1.144 (5)	0.5204 (19)	0.053 (14)*	

### Atomic displacement parameters (Å^2^)

**Table:**

	*U*^11^	*U*^22^	*U*^33^	*U*^12^	*U*^13^	*U*^23^
Ni1	0.0240 (2)	0.0404 (3)	0.0343 (3)	−0.0019 (2)	−0.00076 (18)	0.0032 (2)
Cl3	0.0426 (6)	0.0615 (7)	0.0608 (7)	−0.0183 (5)	−0.0015 (5)	−0.0011 (5)
Cl1	0.0498 (6)	0.0627 (8)	0.0729 (8)	−0.0140 (6)	−0.0032 (5)	0.0246 (6)
Cl4	0.0634 (8)	0.1139 (12)	0.0473 (7)	0.0148 (7)	−0.0164 (6)	0.0139 (7)
Cl2	0.0638 (8)	0.1517 (15)	0.0571 (8)	0.0273 (9)	−0.0192 (6)	0.0263 (8)
O2	0.0269 (13)	0.0485 (16)	0.0427 (15)	−0.0026 (12)	−0.0017 (11)	0.0023 (12)
O1	0.0250 (12)	0.0498 (16)	0.0338 (13)	−0.0039 (11)	−0.0017 (10)	0.0079 (12)
O4	0.0331 (14)	0.0597 (18)	0.0389 (15)	−0.0096 (13)	0.0006 (12)	0.0086 (13)
O6	0.0254 (13)	0.0477 (17)	0.0420 (16)	0.0006 (11)	0.0006 (11)	0.0018 (13)
O3	0.0291 (13)	0.0466 (16)	0.0362 (14)	−0.0065 (11)	−0.0026 (11)	0.0037 (12)
O5	0.0376 (15)	0.0380 (16)	0.0558 (17)	0.0015 (12)	0.0049 (12)	−0.0017 (13)
C1	0.0301 (19)	0.038 (2)	0.0329 (19)	0.0057 (16)	0.0028 (15)	−0.0011 (16)
C7	0.0231 (17)	0.049 (2)	0.046 (2)	−0.0035 (17)	−0.0017 (16)	−0.0043 (19)
C8	0.0279 (18)	0.038 (2)	0.034 (2)	0.0025 (16)	0.0010 (15)	−0.0049 (16)
C9	0.033 (2)	0.041 (2)	0.040 (2)	−0.0006 (17)	0.0021 (16)	−0.0042 (17)
C5	0.031 (2)	0.071 (3)	0.043 (2)	0.010 (2)	−0.0048 (17)	−0.008 (2)
C11	0.042 (2)	0.071 (3)	0.033 (2)	0.010 (2)	−0.0061 (17)	−0.002 (2)
C4	0.042 (2)	0.082 (3)	0.036 (2)	0.018 (2)	−0.0031 (18)	0.007 (2)
C13	0.033 (2)	0.054 (3)	0.035 (2)	−0.0012 (18)	0.0016 (16)	−0.0016 (18)
C12	0.049 (2)	0.059 (3)	0.040 (2)	−0.001 (2)	0.0009 (19)	0.008 (2)
C6	0.0258 (18)	0.047 (2)	0.036 (2)	0.0043 (16)	0.0015 (15)	−0.0056 (17)
C3	0.045 (2)	0.067 (3)	0.047 (2)	0.012 (2)	0.004 (2)	0.016 (2)
C14	0.043 (2)	0.057 (3)	0.038 (2)	−0.011 (2)	0.0053 (18)	0.0128 (19)
C10	0.031 (2)	0.060 (3)	0.044 (2)	0.0008 (19)	−0.0034 (17)	−0.015 (2)
C2	0.038 (2)	0.044 (2)	0.045 (2)	0.0038 (18)	0.0032 (17)	0.0064 (19)
N1	0.056 (2)	0.041 (2)	0.059 (2)	−0.0003 (17)	−0.0052 (18)	−0.0027 (17)
C15	0.040 (2)	0.053 (3)	0.043 (2)	−0.010 (2)	−0.0007 (18)	−0.003 (2)
C16	0.066 (3)	0.075 (4)	0.096 (4)	0.020 (3)	0.015 (3)	−0.009 (3)
C17	0.124 (6)	0.037 (3)	0.142 (6)	−0.015 (3)	−0.008 (5)	0.001 (3)

### Geometric parameters (Å, °)

**Table:**

Ni1—O3	2.041 (2)	C5—C4	1.400 (6)
Ni1—O1	2.041 (2)	C5—C6	1.411 (5)
Ni1—O2	2.061 (3)	C5—H5A	0.9300
Ni1—O4	2.070 (3)	C11—C12	1.372 (6)
Ni1—O5	2.148 (3)	C11—C10	1.426 (6)
Ni1—O6	2.150 (3)	C4—C3	1.408 (6)
Cl3—C9	1.784 (4)	C13—C12	1.416 (5)
Cl1—C2	1.764 (4)	C13—C14	1.448 (5)
Cl4—C11	1.754 (4)	C12—H12A	0.9300
Cl2—C4	1.748 (4)	C3—C2	1.365 (5)
O2—C7	1.239 (4)	C3—H3A	0.9300
O1—C1	1.295 (4)	C14—H14A	0.9300
O4—C14	1.226 (4)	C10—H10A	0.9300
O6—H6A	0.8200	N1—C15	1.356 (5)
O6—H6B	0.83 (4)	N1—C16	1.456 (6)
O3—C8	1.292 (4)	N1—C17	1.534 (6)
O5—C15	1.301 (5)	C15—H15A	0.9300
C1—C6	1.437 (5)	C16—H16A	0.9600
C1—C2	1.463 (5)	C16—H16B	0.9600
C7—C6	1.483 (5)	C16—H16C	0.9600
C7—H7A	0.9300	C17—H17A	0.9600
C8—C9	1.441 (5)	C17—H17B	0.9600
C8—C13	1.465 (5)	C17—H17C	0.9600
C9—C10	1.374 (5)		
			
O3—Ni1—O1	92.08 (10)	C5—C4—Cl2	121.1 (4)
O3—Ni1—O2	177.03 (10)	C3—C4—Cl2	118.0 (3)
O1—Ni1—O2	90.12 (10)	C12—C13—C14	114.5 (4)
O3—Ni1—O4	90.51 (10)	C12—C13—C8	122.9 (3)
O1—Ni1—O4	176.71 (10)	C14—C13—C8	122.7 (3)
O2—Ni1—O4	87.36 (10)	C11—C12—C13	119.2 (4)
O3—Ni1—O5	92.13 (10)	C11—C12—H12A	120.4
O1—Ni1—O5	88.33 (10)	C13—C12—H12A	120.4
O2—Ni1—O5	89.92 (10)	C5—C6—C1	119.4 (4)
O4—Ni1—O5	89.55 (11)	C5—C6—C7	116.9 (3)
O3—Ni1—O6	92.88 (10)	C1—C6—C7	123.7 (3)
O1—Ni1—O6	95.38 (10)	C2—C3—C4	118.5 (4)
O2—Ni1—O6	84.93 (10)	C2—C3—H3A	120.8
O4—Ni1—O6	86.51 (11)	C4—C3—H3A	120.8
O5—Ni1—O6	173.65 (10)	O4—C14—C13	127.9 (4)
C7—O2—Ni1	123.9 (2)	O4—C14—H14A	116.0
C1—O1—Ni1	126.3 (2)	C13—C14—H14A	116.0
C14—O4—Ni1	125.1 (2)	C9—C10—C11	121.5 (4)
Ni1—O6—H6A	109.5	C9—C10—H10A	119.3
Ni1—O6—H6B	106 (3)	C11—C10—H10A	119.3
H6A—O6—H6B	96.5	C3—C2—C1	123.6 (4)
C8—O3—Ni1	124.5 (2)	C3—C2—Cl1	117.6 (3)
C15—O5—Ni1	126.0 (2)	C1—C2—Cl1	118.9 (3)
O1—C1—C6	123.1 (3)	C15—N1—C16	118.1 (4)
O1—C1—C2	120.6 (3)	C15—N1—C17	124.0 (4)
C6—C1—C2	116.3 (3)	C16—N1—C17	117.4 (4)
O2—C7—C6	128.0 (3)	O5—C15—N1	127.4 (4)
O2—C7—H7A	116.0	O5—C15—H15A	116.3
C6—C7—H7A	116.0	N1—C15—H15A	116.3
O3—C8—C9	119.8 (3)	N1—C16—H16A	109.5
O3—C8—C13	125.9 (3)	N1—C16—H16B	109.5
C9—C8—C13	114.4 (3)	H16A—C16—H16B	109.5
C10—C9—C8	121.9 (4)	N1—C16—H16C	109.5
C10—C9—Cl3	119.8 (3)	H16A—C16—H16C	109.5
C8—C9—Cl3	118.3 (3)	H16B—C16—H16C	109.5
C4—C5—C6	121.4 (4)	N1—C17—H17A	109.5
C4—C5—H5A	119.3	N1—C17—H17B	109.5
C6—C5—H5A	119.3	H17A—C17—H17B	109.5
C12—C11—C10	120.1 (4)	N1—C17—H17C	109.5
C12—C11—Cl4	119.0 (3)	H17A—C17—H17C	109.5
C10—C11—Cl4	120.9 (3)	H17B—C17—H17C	109.5
C5—C4—C3	120.9 (4)		

### Hydrogen-bond geometry (Å, °)

**Table:**

*D*—H···*A*	*D*—H	H···*A*	*D*···*A*	*D*—H···*A*
O6—H6A···O1^i^	0.82	1.91	2.714 (3)	168
O6—H6B···O3^i^	0.83 (4)	2.17 (4)	2.850 (4)	139 (4)
O6—H6B···Cl3^i^	0.84 (4)	2.67 (4)	3.374 (3)	143 (4)

Symmetry codes: (i) −*x*, −*y*+2, −*z*+1.
